# Supplementary material for: Genome-Wide Identification and Expression Analysis of GA2ox, GA3ox, and GA20ox Are Related to Gibberellin Oxidase Genes in Grape (Vitis vinifera L.)
Source: Genes (Basel). 2019 Sep 5;10(9):680. doi: 10.3390/genes10090680 (PMC6771001; doi:10.3390/genes10090680)
Supplement: Supplementary file 1 [file genes-10-00680-s001.zip › Table S1.docx]

Table S1.GA oxidase genes in Arabidopsis, Rice, Apples and Grapes.

| Gene name | Entry ID | Chromosome | Site |
| --- | --- | --- | --- |
| AtGA2ox1 | At1g78440 | 1 | 29511772-29512990 |
| AtGA2ox2 | At1g30040 | 1 | 10537769-10539570 |
| AtGA2ox3 | At2g34555 | 2 | 14557102-14558682 |
| AtGA2ox4 | At1g47990 | 1 | 17698655-17700834 |
| AtGA2ox6 | At1g02400 | 1 | 486964-489391 |
| AtGA2ox7 | At1g50960 | 1 | 18889549-18891719 |
| AtGA2ox8 | At4g21200 | 4 | 11302751-11306601 |
| AtGA3ox1 | At1g15550 | 1 | 5344569-5346078 |
| AtGA3ox2 | At1g80340 | 1 | 30200695-30202163 |
| AtGA3ox3 | At4g21690 | 4 | 11527229-11529060 |
| AtGA3ox4 | At1g80330 | 1 | 30198061-30199537 |
| AtGA20ox1 | At4g25420 | 4 | 12990982-12992409 |
| AtGA20ox2 | At5g51810 | 5 | 21055389-21056746 |
| AtGA20ox3 | At5g07200 | 5 | 2243835-2245157 |
| AtGA20ox4 | At1g60980 | 1 | 22452573-22454140 |
| AtGA20ox5 | At1g44090 | 1 | 16760677-16762486 |
| OsGA2ox1 | Os05g06670 | 5 | 3459593-3465968 |
| OsGA2ox2 | Os01g22920 | 1 | 12884525-12883448 |
| OsGA2ox3 | Os01g55240 | 1 | 31794061-31796599 |
| OsGA2ox4 | Os05g43880 | 5 | 25453625-25452474 |
| OsGA2ox5 | Os07g01340 | 7 | 212778-218544 |
| OsGA2ox6 | Os04g44150 | 4 | 25949230-25945581 |
| OsGA2ox7 | Os01g11150 | 1 | 5971489-5967819 |
| OsGA2ox8 | Os05g48700 | 5 | 27850281-27848088 |
| OsGA2ox9 | Os02g41954 | 2 | 25197873-25193517 |
| OsGA2ox10 | Os05g11810 | 5 | 6726915-6720940 |
| OsGA2ox11 | Os04g33360 | 4 | 20028114-20029927 |
| OsGA3ox1 | Os05g08540 | 5 | 4662566-4660639 |
| OsGA3ox2 | Os01g08220 | 1 | 4003946-4002659 |
| OsGA20ox1 | Os03g63970 | 3 | 36143590-36145443 |
| OsGA20ox2 | Os01g66100 | 1 | 38381339-38384461 |
| OsGA20ox3 | Os07g07420 | 7 | 3701288-3704031 |
| OsGA20ox4 | Os05g34854 | 5 | 20629715-20622787 |
| OsGA20ox5 | Os03g42130 | 3 | 23450345-23448338 |
| OsGA20ox6 | Os04g39980 | 4 | 23612388-23610266 |
| OsGA20ox7 | Os08g44590 | 8 | 28040495-28042874 |
| OsGA20ox8 | Os04g55070 | 4 | 32565717-32562078 |
| MdGA2ox1 | MDP0000155229 | 1 | 28963756-28965591 |
| MdGA2ox2 | MDP0000309451 | 5 | 1669133-1674438 |
| MdGA2ox3 | MDP0000177939 | 5 | 1676527-1679342 |
| MdGA2ox4 | MDP0000247490 | 5 | 6628238-6629873 |
| MdGA2ox5 | MDP0000322440 | 5 | 6655126-6656761 |
| MdGA2ox6 | MDP0000185333 | 5 | 14153513-14155382 |
| MdGA2ox7 | MDP0000155054 | 9 | 31362315-31365362 |
| MdGA2ox8 | MDP0000145841 | 9 | 31364217-31366325 |
| MdGA2ox9 | MDP0000137705 | 10 | 19867370-19869026 |
| MdGA2ox10 | MDP0000145827 | 10 | 25855432-25857023 |
| MdGA2ox11 | MDP0000166016 | 11 | 6286163-6288020 |
| MdGA2ox12 | MDP0000132878 | 13 | 466337-467008 |
| MdGA2ox13 | MDP0000277073 | 13 | 512336-512788 |
| MdGA2ox14 | MDP0000226405 | 13 | 12250450-12255706 |
| MdGA2ox15 | MDP0000192372 | 13 | 12263428-12264270 |
| MdGA2ox16 | MDP0000139968 | 14 | 23716455-23717242 |
| MdGA2ox17 | MDP0000269990 | 16 | 7343103-7344582 |
| MdGA2ox18 | MDP0000735747 | 16 | 8036240-8040685 |
| MdGA2ox19 | MDP0000161181 | 17 | 24286837-24290755 |
| MdGA2ox20 | MDP0000875320 | unanchored | 14027945-14028331 |
| MdGA3ox1 | MDP0000233590 | 2 | 30310394-30311669 |
| MdGA3ox2 | MDP0000424553 | 2 | 30314053-30315559 |
| MdGA3ox3 | MDP0000297548 | 3 | 828254-828997 |
| MdGA3ox4 | MDP0000316943 | 7 | 4325975-4327310 |
| MdGA3ox5 | MDP0000381531 | 8 | 6696717-6705944 |
| MdGA3ox6 | MDP0000172806 | 8 | 11327232-11328069 |
| MdGA3ox7 | MDP0000716315 | 9 | 21448680-21457474 |
| MdGA3ox8 | MDP0000822659 | 9 | 21448936-21449287 |
| MdGA3ox9 | MDP0000195618 | 12 | 13232577-13234575 |
| MdGA3ox10 | MDP0000262840 | 12 | 13233698-13235968 |
| MdGA3ox11 | MDP0000130015 | 15 | 5037601-5039021 |
| MdGA3ox12 | MDP0000239572 | 15 | 11469511-11471069 |
| MdGA3ox13 | MDP0000320360 | 15 | 11474700-11476696 |
| MdGA3ox14 | MDP0000238083 | unanchored | 96167171-96167731 |
| MdGA20ox1 | MDP0000248981 | 1 | 24897671-24899153 |
| MdGA20ox2 | MDP0000136940 | 2 | 21725795-21727282 |
| MdGA20ox3 | MDP0000127230 | 6 | 6482041-6483684 |
| MdGA20ox4 | MDP0000234714 | 6 | 6487233-6490435 |
| MdGA20ox5 | MDP0000280240 | 7 | 23081984-23083448 |
| MdGA20ox6 | MDP0000128715 | 11 | 18766444-18767915 |
| MdGA20ox7 | MDP0000136905 | 15 | 3027308-3030896 |
| VvGA2ox1 | GSVIVT01000687001 | 19 | 15497729-15499650 |
| VvGA2ox2 | GSVIVT01000689001 | 19 | 15603207-15604930 |
| VvGA2ox3 | GSVIVT01001966001 | 19 | 5864698-5871371 |
| VvGA2ox4 | GSVIVT01010228001 | 1 | 17965019-17966964 |
| VvGA2ox5 | GSVIVT01012628001 | 10 | 190065-195087 |
| VvGA2ox6 | GSVIVT01015671001 | 3 | 15307070-15312521 |
| VvGA2ox7 | GSVIVT01021468001 | 10 | 5846741-5848388 |
| VvGA2ox8 | GSVIVT01028169001 | 7 | 4377203-4380857 |
| VvGA2ox9 | GSVIVT01031814001 | 3 | 4642801-4644279 |
| VvGA2ox10 | GSVIVT01031826001 | 3 | 4723791-4725151 |
| VvGA2ox11 | GSVIVT01034945001 | 5 | 343990-346779 |
| VvGA3ox1 | GSVIVT01008811001 | 18 | 2346915-2348502 |
| VvGA3ox2 | GSVIVT01017173001 | 9 | 4993413-4995290 |
| VvGA3ox3 | GSVIVT01017178001 | 9 | 5037569-5040878 |
| VvGA3ox4 | GSVIVT01020680001 | 12 | 3145880-3152445 |
| VvGA3ox5 | GSVIVT01026928001 | 15 | 19336280-19337354 |
| VvGA3ox6 | GSVIVT01035796001 | 4 | 4431413-4432859 |
| VvGA20ox1 | GSVIVT01008782001 | 18 | 1982673-1985445 |
| VvGA20ox2 | GSVIVT01018453001 | 16 | 14861395-14863190 |
| VvGA20ox3 | GSVIVT01019696001 | 2 | 2527135-2532638 |
| VvGA20ox4 | GSVIVT01026453001 | 4 | 23382368-23384135 |
| VvGA20ox5 | GSVIVT01026466001 | 4 | 23110638-23112150 |
| VvGA20ox6 | GSVIVT01027572001 | 15 | 15453533-15455130 |
| VvGA20ox7 | GSVIVT01031837001 | 3 | 4782181-4783671 |
