# Supplementary material for: Genome-Wide Identification and Expression Analysis of GA2ox, GA3ox, and GA20ox Are Related to Gibberellin Oxidase Genes in Grape (Vitis vinifera L.)
Source: Genes (Basel). 2019 Sep 5;10(9):680. doi: 10.3390/genes10090680 (PMC6771001; doi:10.3390/genes10090680)
Supplement: Supplementary file 1 [file genes-10-00680-s001.zip › Table S2.docx]

Table S2. Oligonucleotides used for qRT-PCR of GA2ox,GA3ox and GA20ox gene family in grape

| Gene | Sequence of oligonucleotides forward (F) and reverse (R) | |
| --- | --- | --- |
| VvGA2ox1 | F  R | 5’-TTGGATCAAGCGGCGATATTGGTC-3’  5’-CTCACAAGCCATCTTCCTCACAGC-3’ |
| VvGA2ox3 | F  R | 5’-TGGCAACTTGGAGCATGAAGAGTG-3’  5’-TGGAATGGCTGCCGGAACAAC-3’ |
| VvGA2ox4 | F  R | 5’-GCAGTTCAGCAACGTGATTGTGG-3’  5’-AGCCTCTCCTCTTCACTCTCACTC-3’ |
| VvGA2ox5 | F  R | 5’-TTCGCTTGAAGACCAACCACCAG-3’  5’-GGAGGCCACCGGAAGAGGAG-3’ |
| VvGA2ox6 | F  R | 5’-TGCCAACTTGTATCCACCTTGTCC-3’  5’-AGGCCGCCAACTTCATTCTGTG-3’ |
| VvGA2ox7 | F  R | 5’-TTATCGCAGAATGGTCGTCAGCAG-3’  5’-CCATGTGAGCCTTGGCGTGAG-3’ |
| VvGA2ox8 | F  R | 5’-CCAGTTCGGAGAAGCAGAAGGC-3’  5’-GGAGACAGGATTCGTGTGGAGAAG-3’ |
| VvGA2ox9 | F  R | 5’-AGAGGTCGATCCAGCCTTCATCC-3’  5’-TGGTTGGAAGCATTGGCGGAAG-3’ |
| VvGA2ox10 | F  R | 5’-CGATCCAGCCTTCATCCAAGACAC-3’  5’-TGGTTGGAAGCATTGGCGGAAG-3’ |
| VvGA2ox11 | F  R | 5’-TGGTGGTGGCCTCTCCAACC-3’  5’-CACTATCAGCTCTGCTACCTTCGG-3’ |
| VvGA3ox1 | F  R | 5’-GGCTCGGATCGCTAATGGATGC-3’  5’-TTCCACCAACTCGTTCAGCAACC-3’ |
| VvGA3ox2 | F  R | 5’-GCAGCCTCCTCGACGACATTG-3’  5’-GTGGAGATCCGAAGATGGTGAAGC-3’ |
| VvGA3ox4 | F  R | 5’-CACACGCGACTTCAAGGACAAGG-3’  5’-TCAGGAGGATTCGGAACCATAGGC-3’ |
| VvGA3ox5 | F  R | 5’-AGCCTCAGGGTTTCTGGACC-3’  5’-CACCCAAGCTCATCAGCACA-3’ |
| VvGA3ox6 | F  R | 5’-CCTGGACTTCACCTCCGTTCATTC-3’  5’-GCCAACAAGCTGAGCAGCATTG-3’ |
| VvGA20ox1 | F  R | 5’-AGCCGGTGGTGGATCTTGAGG-3’  5’-ATCACTTGGAAGAAGCCGTGGTTC-3’ |
| VvGA20ox2 | F  R | 5’-AAGTCATCCAATGCTGTGCAGGAG-3’  5’-TGCTCATGGCCTCACAGTAGTCC-3’ |
| VvGA20ox3 | F  R | 5’-TCCAATCCAGTCAGGCTCTCCAC-3’  5’-GGTGCAATACTCAGCCACATCCTC-3’ |
| VvGA20ox4 | F  R | 5’-AAGGTGATGAGGCTGCAACTGC-3’  5’-TTCGCCAGGCTTCTTCTTGACAC-3’ |
| VvGA20ox5 | F  R | 5’-CCGTGCTACAGAGGTTGGATTGG-3’  5’-GGCACCACAGTATCCGCATAACTC-3’ |
| VvGA20ox6 | F  R | 5’-GATCAGGTGTCTGGCCTTCAAGTG-3’  5’-GCCATGAATGTGTCACCGATGTTG-3’ |
| VvGA20ox7 | F  R | 5’-TCGCTGTTATCGAAGCTGAAGGC-3’  5’-GAAGAACTTCCTCGCGGCATCC-3’ |
| GAPDH | F | 5'-TTCTCGTTGAGGGCTATTCCA-3' |
|  | R | 5'-CCACAGACTTCATCGGTGACA-3' |
