# Supplementary material for: Genome-Wide Identification and Expression Analysis of GA2ox, GA3ox, and GA20ox Are Related to Gibberellin Oxidase Genes in Grape (Vitis vinifera L.)
Source: Genes (Basel). 2019 Sep 5;10(9):680. doi: 10.3390/genes10090680 (PMC6771001; doi:10.3390/genes10090680)
Supplement: Supplementary file 1 [file genes-10-00680-s001.zip › Table S3.docx]

Table S3. Motif sequences of GA oxidase in grape

| Motif number | E-value | Sites | Consensus sequence |
| --- | --- | --- | --- |
| motif1 | 9.6e-527 | 24 | QVLKBGKWISVKPNPDALVINIGDLLQALSNGRYKSVLHRVVVNSEKERF |
| motif2 | 5.7e-230 | 24 | GLGPHTDPQLLTILHQDQVGGL |
| motif3 | 1.2e-224 | 22 | AELIAEACEEWGFFQVINHGVPSEL |
| motif4 | 3.3e-198 | 24 | WPZDPEEFREVVZEYAKAMKKLALKJLELJAESLGLEKNYF |
| motif5 | 5.6e-134 | 23 | FEESDSVLRLNHYPPCPEPEL |
| motif6 | 1.4e-088 | 22 | SLAYFLCPPLDKVISPLPELV |
| motif7 | 1.1e-067 | 15 | IRKJEDEARKFFALPLSEKLKAGRPPPFG |
| motif8 | 8.2e-045 | 23 | EELSIPLIDLSGPNS |
| motif9 | 2.4e-038 | 16 | YRKFTWGEYKQTVYK |
| motif10 | 1.4e-029 | 3 | GYFDSELTKNVRDWKEVFDFVVSTPTVIPVSPDPDDKELKE |
