# Supplementary material for: Genome-Wide Identification and Expression Analysis of GA2ox, GA3ox, and GA20ox Are Related to Gibberellin Oxidase Genes in Grape (Vitis vinifera L.)
Source: Genes (Basel). 2019 Sep 5;10(9):680. doi: 10.3390/genes10090680 (PMC6771001; doi:10.3390/genes10090680)
Supplement: Supplementary file 1 [file genes-10-00680-s001.zip › Table S4.docx]

Table S4: Codon usage characteristics of three gibberellin oxidase gene families in grape

| Gene | T3s | C3s | A3s | G3s | CAI | CBI | Fop | Nc | GC3s | GC | L_sym | L_aa | Gravy | Aromo |
| --- | --- | --- | --- | --- | --- | --- | --- | --- | --- | --- | --- | --- | --- | --- |
| *VvGA2ox1* | 0.3525 | 0.2989 | 0.3361 | 0.2752 | 0.187 | -0.032 | 0.394 | 49.39 | 0.445 | 0.445 | 310 | 323 | -0.193499 | 0.099071 |
| *VvGA2ox2* | 0.3396 | 0.3022 | 0.324 | 0.2969 | 0.178 | -0.048 | 0.383 | 53.44 | 0.464 | 0.438 | 321 | 332 | -0.210843 | 0.096386 |
| *VvGA2ox3* | 0.4055 | 0.2638 | 0.3471 | 0.3077 | 0.218 | -0.029 | 0.407 | 53.83 | 0.419 | 0.428 | 322 | 329 | -0.304255 | 0.112462 |
| *VvGA2ox4* | 0.3872 | 0.2458 | 0.3552 | 0.2996 | 0.196 | -0.067 | 0.382 | 49.9 | 0.417 | 0.459 | 374 | 390 | -0.381026 | 0.094872 |
| *VvGA2ox5* | 0.3242 | 0.3639 | 0.3032 | 0.2941 | 0.233 | 0.071 | 0.458 | 54.39 | **0.505** | 0.466 | 404 | 421 | -0.245368 | 0.114014 |
| *VvGA2ox6* | 0.3262 | 0.3548 | 0.3011 | 0.2749 | 0.194 | -0.055 | 0.376 | 53.68 | 0.494 | 0.47 | 340 | 352 | -0.244886 | 0.076705 |
| *VvGA2ox7* | 0.3118 | 0.3346 | 0.256 | 0.3766 | 0.219 | 0.047 | 0.439 | 50.1 | **0.545** | 0.486 | 321 | 333 | -0.287688 | 0.09009 |
| *VvGA2ox8* | 0.3058 | 0.3417 | 0.2609 | 0.3261 | 0.156 | -0.118 | 0.333 | 51.05 | **0.53** | 0.497 | 321 | 334 | -0.129641 | 0.083832 |
| *VvGA2ox9* | 0.3496 | 0.3383 | 0.2421 | 0.3793 | 0.204 | -0.041 | 0.392 | 55.22 | **0.536** | 0.481 | 332 | 342 | -0.519883 | 0.105263 |
| *VvGA2ox10* | 0.3496 | 0.3346 | 0.2412 | 0.3739 | 0.196 | -0.032 | 0.396 | 53.94 | **0.535** | 0.482 | 333 | 342 | -0.540058 | 0.102339 |
| *VvGA2ox11* | 0.3381 | 0.3203 | 0.2778 | 0.319 | 0.204 | -0.039 | 0.392 | 51.94 | 0.498 | 0.486 | 329 | 339 | -0.212979 | 0.088496 |
| *VvGA3ox1* | 0.2513 | 0.407 | 0.2512 | 0.4263 | **0.265** | 0.072 | 0.46 | **45.88** | **0.616** | **0.504** | 263 | 274 | -0.458029 | 0.091241 |
| *VvGA3ox2* | 0.2736 | 0.4495 | 0.237 | 0.25 | 0.226 | 0.087 | 0.457 | 53.64 | **0.577** | **0.536** | 350 | 365 | -0.131507 | 0.087671 |
| *VvGA3ox3* | 0.3333 | 0.4167 | 0.2509 | 0.2205 | 0.249 | 0.086 | 0.456 | **57.27** | **0.517** | **0.515** | 344 | 359 | -0.040668 | 0.083565 |
| *VvGA3ox4* | 0.367 | 0.2532 | 0.3618 | 0.2827 | 0.189 | -0.079 | 0.365 | 56.26 | 0.416 | 0.453 | 654 | 678 | -0.245575 | 0.08997 |
| *VvGA3ox5* | 0.4118 | 0.2431 | 0.332 | 0.3009 | 0.235 | 0.054 | 0.451 | 46.88 | 0.413 | 0.431 | 315 | 328 | -0.216768 | 0.112805 |
| *VvGA3ox6* | 0.3797 | 0.3458 | 0.2538 | 0.247 | 0.202 | -0.012 | 0.401 | 56.67 | 0.477 | 0.496 | 342 | 355 | -0.174648 | 0.092958 |
| *VvGA20ox1* | 0.4018 | 0.2931 | 0.331 | 0.2509 | 0.227 | -0.012 | 0.413 | 53.4 | 0.421 | 0.446 | 392 | 410 | -0.360732 | 0.112195 |
| *VvGA20ox2* | 0.3248 | 0.3504 | 0.3173 | 0.2929 | 0.236 | 0.043 | 0.447 | 50.9 | 0.496 | 0.477 | 282 | 296 | -0.224324 | 0.108108 |
| *VvGA20ox3* | 0.3419 | 0.3493 | 0.2641 | 0.2944 | 0.209 | 0.013 | 0.407 | 56.71 | **0.506** | 0.483 | 312 | 321 | -0.276636 | 0.077882 |
| *VvGA20ox4* | 0.375 | 0.3125 | 0.293 | 0.3127 | 0.229 | 0.007 | 0.423 | 55.69 | 0.476 | 0.462 | 359 | 377 | -0.409549 | 0.103448 |
| *VvGA20ox5* | 0.4276 | 0.2897 | 0.3498 | 0.245 | 0.233 | -0.036 | 0.404 | 53.93 | 0.402 | 0.43 | 361 | 375 | -0.3448 | 0.114667 |
| *VvGA20ox6* | 0.3713 | 0.3249 | 0.3301 | 0.2538 | 0.19 | -0.073 | 0.373 | 54.45 | 0.447 | 0.457 | 284 | 298 | -0.267785 | 0.104027 |
| *VvGA20ox7* | 0.351 | 0.3592 | 0.2533 | 0.3349 | 0.215 | -0.017 | 0.403 | 54.28 | **0.525** | 0.487 | 303 | 312 | -0.418269 | 0.102564 |
