# Supplementary material for: Genome-Wide Identification and Expression Analysis of GA2ox, GA3ox, and GA20ox Are Related to Gibberellin Oxidase Genes in Grape (Vitis vinifera L.)
Source: Genes (Basel). 2019 Sep 5;10(9):680. doi: 10.3390/genes10090680 (PMC6771001; doi:10.3390/genes10090680)
Supplement: Supplementary file 1 [file genes-10-00680-s001.zip › Table S5.docx]

Table S5: Correlation coefficient between the parameters of codon usage of three gibberellin oxidase gene families in grape

|  | T3s | C3s | A3s | G3s | CAI | CBI | Fop | Nc | GC3s | GC | L_sym | L_aa | Gravy |
| --- | --- | --- | --- | --- | --- | --- | --- | --- | --- | --- | --- | --- | --- |
| C3s | **-0.783**** |  |  |  |  |  |  |  |  |  |  |  |  |
| A3s | **0.635**** | **-0.783**** |  |  |  |  |  |  |  |  |  |  |  |
| G3s | **-0.407*** | 0.053 | -0.398 |  |  |  |  |  |  |  |  |  |  |
| CAI | -0.091 | 0.333 | -0.133 | 0.053 |  |  |  |  |  |  |  |  |  |
| CBI | -0.362 | **0.545**** | -0.36 | 0.018 | **0.842**** |  |  |  |  |  |  |  |  |
| Fop | -0.255 | **0.434*** | -0.243 | 0.043 | **0.900**** | **0.980**** |  |  |  |  |  |  |  |
| Nc | 0.227 | 0.159 | -0.141 | **-0.459*** | -0.159 | -0.141 | -0.203 |  |  |  |  |  |  |
| GC3s | **-0.892**** | **0.851**** | **-0.885**** | **0.548**** | 0.217 | **0.430*** | 0.332 | -0.107 |  |  |  |  |  |
| GC | **-0.760**** | **0.865**** | **-0.857**** | 0.157 | 0.158 | 0.39 | 0.263 | 0.118 | **0.847**** |  |  |  |  |
| L_sym | 0.229 | -0.344 | 0.377 | -0.248 | -0.167 | -0.229 | -0.229 | 0.371 | -0.376 | -0.187 |  |  |  |
| L_aa | 0.227 | -0.339 | 0.383 | -0.26 | -0.158 | -0.22 | -0.22 | 0.365 | -0.379 | -0.185 | **0.999**** |  |  |
| Gravy | -0.151 | 0.193 | 0.078 | **-0.648**** | -0.126 | 0.153 | 0.053 | 0.089 | -0.103 | 0.185 | 0.07 | 0.079 |  |
| Aromo | **0.567**** | **-0.422*** | **0.437*** | -0.008 | 0.296 | 0.073 | 0.234 | -0.119 | **-0.445*** | **-0.604**** | -0.038 | -0.034 | -0.378 |
