# Supplementary material for: Genome-Wide Identification and Expression Analysis of GA2ox, GA3ox, and GA20ox Are Related to Gibberellin Oxidase Genes in Grape (Vitis vinifera L.)
Source: Genes (Basel). 2019 Sep 5;10(9):680. doi: 10.3390/genes10090680 (PMC6771001; doi:10.3390/genes10090680)
Supplement: Supplementary file 1 [file genes-10-00680-s001.zip › Table S6.docx]

Table S6: Frequency of use of three grape gibberellin oxidase gene synonymous codons

| Amino acid | Codon | The number of codon | | | RSCU | | |
| --- | --- | --- | --- | --- | --- | --- | --- |
|  |  | VvGA2oxs | VvGA3oxs | VvGA20oxs | VvGA2oxs | VvGA3oxs | VvGA20oxs |
| Phe | UUU | 8 | 8 | 8 | 0.80 | 0.72 | 0.76 |
|  | UUC | 12 | 11 | 12 | **1.20** | **1.28** | **1.24** |
| Leu | UUA | 4 | 5 | 4 | 0.67 | 0.58 | 0.78 |
|  | UUG | 7 | 7 | 6 | 1.33 | 0.98 | 0.98 |
|  | CUU | 6 | 9 | 8 | **1.15** | **1.16** | **1.49** |
|  | CUC | 7 | 11 | 6 | **1.35** | **1.59** | **1.09** |
|  | CUA | 2 | 4 | 4 | 0.42 | 0.54 | 0.74 |
|  | CUG | 6 | 7 | 5 | 1.08 | 1.15 | 0.91 |
| Ile | AUU | 8 | 4 | 5 | 1.25 | 0.66 | 1.14 |
|  | AUC | 7 | 9 | 5 | **1.07** | **1.55** | **1.11** |
|  | AUA | 4 | 5 | 3 | 0.68 | 0.79 | 0.76 |
| Met | AUG | 7 | 9 | 9 | **1.00** | **1.00** | **1.00** |
| Val | GUU | 8 | 10 | 8 | **1.33** | **1.33** | **1.37** |
|  | GUC | 5 | 5 | 4 | 0.77 | 0.76 | 0.70 |
|  | GUA | 2 | 5 | 3 | 0.28 | 0.56 | 0.46 |
|  | GUG | 10 | 10 | 9 | **1.62** | **1.35** | **1.47** |
| Ser | UCU | 7 | 8 | 5 | **1.39** | **1.60** | **1.34** |
|  | UCC | 5 | 5 | 4 | **1.07** | **1.01** | **1.00** |
|  | UCA | 6 | 6 | 7 | **1.26** | **1.04** | **1.60** |
|  | UCG | 2 | 2 | 1 | 0.47 | 0.45 | 0.28 |
| Pro | CCU | 8 | 10 | 8 | **1.39** | **1.52** | **1.39** |
|  | CCC | 5 | 5 | 5 | 0.77 | 0.75 | 0.79 |
|  | CCA | 9 | 8 | 8 | **1.52** | **1.31** | **1.45** |
|  | CCG | 2 | 3 | 2 | 0.32 | 0.42 | 0.36 |
| Thr | ACU | 4 | 6 | 4 | **1.04** | **1.23** | **1.31** |
|  | ACC | 4 | 6 | 4 | **1.23** | **1.67** | **1.37** |
|  | ACA | 5 | 4 | 4 | 1.36 | 0.90 | 1.13 |
|  | ACG | 1 | 1 | 1 | 0.37 | 0.20 | 0.18 |
| Ala | GCU | 7 | 10 | 7 | **1.63** | **1.81** | **1.68** |
|  | GCC | 4 | 6 | 5 | 0.99 | 0.82 | 1.22 |
|  | GCA | 5 | 7 | 4 | 1.14 | 1.08 | 0.87 |
|  | GCG | 1 | 2 | 1 | 0.24 | 0.29 | 0.22 |
| Tyr | UAU | 4 | 4 | 5 | 0.91 | 0.74 | 0.87 |
|  | UAC | 5 | 7 | 6 | **1.09** | **1.26** | **1.13** |
| TER | UAA | 0 | 0 | 1 | 0.82 | 1.00 | 2.14 |
|  | UAG | 0 | 0 | 0 | 0.82 | 0.50 | 0.43 |
| His | CAU | 5 | 7 | 7 | **1.07** | **1.08** | **1.30** |
|  | CAC | 4 | 5 | 4 | 0.93 | 0.93 | 0.70 |
| Gln | CAA | 6 | 8 | 7 | 0.85 | **1.05** | **1.12** |
|  | CAG | 8 | 6 | 6 | 1.15 | 0.95 | 0.88 |
| Asn | AAU | 8 | 7 | 6 | 0.97 | 0.86 | 0.89 |
|  | AAC | 8 | 8 | 8 | **1.03** | **1.14** | **1.11** |
| Lys | AAA | 8 | 9 | 9 | 0.79 | 0.72 | 0.82 |
|  | AAG | 12 | 15 | 12 | **1.21** | **1.28** | **1.18** |
| Asp | GAU | 9 | 11 | 13 | **1.07** | **1.05** | **1.26** |
|  | GAC | 7 | 11 | 8 | 0.93 | 0.95 | 0.74 |
| Glu | GAA | 12 | 12 | 11 | 0.88 | 0.96 | 0.93 |
|  | GAG | 15 | 13 | 12 | **1.12** | **1.04** | **1.07** |
| Cys | UGU | 4 | 3 | 4 | 1.12 | 0.99 | 0.88 |
|  | UGC | 3 | 3 | 4 | 0.88 | **1.02** | **1.12** |
| TER | UGA | 0 | 1 | 0 | 1.36 | 1.50 | 0.43 |
| Trp | UGG | 5 | 6 | 5 | **1.00** | **1.00** | **1.00** |
| Arg | CGU | 1 | 1 | 1 | 0.25 | 0.44 | 0.48 |
|  | CGC | 2 | 3 | 1 | 0.55 | 0.97 | 0.43 |
|  | CGA | 1 | 1 | 1 | 0.35 | 0.51 | 0.49 |
|  | CGG | 1 | 2 | 1 | 0.31 | 0.86 | 0.31 |
| Ser | AGU | 5 | 5 | 4 | 0.96 | 0.86 | 0.81 |
|  | AGC | 4 | 5 | 4 | 0.86 | 1.04 | 0.96 |
| Arg | AGA | 6 | 4 | 4 | **2.04** | **1.21** | **1.73** |
|  | AGG | 7 | 4 | 6 | **2.51** | **2.02** | **2.57** |
| Gly | GGU | 4 | 6 | 5 | 0.68 | 0.86 | 0.99 |
|  | GGC | 5 | 9 | 5 | 0.89 | **1.36** | **1.16** |
|  | GGA | 7 | 8 | 4 | 1.38 | 1.10 | 0.90 |
|  | GGG | 6 | 5 | 4 | 1.05 | 0.68 | 0.95 |
