# Supplementary material for: Genome-Wide Identification and Expression Analysis of GA2ox, GA3ox, and GA20ox Are Related to Gibberellin Oxidase Genes in Grape (Vitis vinifera L.)
Source: Genes (Basel). 2019 Sep 5;10(9):680. doi: 10.3390/genes10090680 (PMC6771001; doi:10.3390/genes10090680)
Supplement: Supplementary file 1 [file genes-10-00680-s001.zip › Table S7.docx]

Table S7. Number of cis-acting elements in GA2ox，GA3ox and GA20ox promoter of grape

| Gene name | MeJA-related | Stress- related | Gibberellin- related | Salicylic  acid-related | | Auxin-related | Abscisic  acid-related | Meristem-related | Circadian-related |
| --- | --- | --- | --- | --- | --- | --- | --- | --- | --- |
| *VvGA2ox1* | 4 | 2 | 1 | | 1 | — | 8 | — | — |
| *VvGA2ox2* | 4 | 2 | — | | — | 1 | 3 | 1 | 1 |
| *VvGA2ox3* | — | 1 | — | | 1 | — | — | — | — |
| *VvGA2ox4* | 6 | 4 | 1 | | 3 | — | 1 | — | — |
| *VvGA2ox5* | 1 | 2 | — | | 2 | — | 5 | — | — |
| *VvGA2ox6* | 4 | 1 | 3 | | — | 2 | 3 | — | 1 |
| *VvGA2ox7* | — | 1 | 2 | | — | — | 1 | — | — |
| *VvGA2ox8* | — | — | 1 | | 2 | — | 1 | — | — |
| *VvGA2ox9* | 4 | 1 | — | | — | — | — | — | — |
| *VvGA2ox10* | 2 | 2 | 1 | | — | — | — | — | — |
| *VvGA2ox11* | — | — | 1 | | — | — | 3 | — | — |
| *VvGA3ox1* | 4 | 1 | 1 | | — | — | 3 | 2 | — |
| *VvGA3ox2* | — | 1 | 1 | | 4 | — | 4 | — | — |
| *VvGA3ox3* | — | — | 1 | | — | — | 4 | — | — |
| *VvGA3ox4* | 6 | — | 2 | | — | — | 1 | — | — |
| *VvGA3ox5* | 2 | 1 | 1 | | — | — | — | — | — |
| *VvGA3ox6* | — | 1 | — | | 4 | — | 3 | 1 | 1 |
| *VvGA20ox1* | 4 | 3 | — | | — | 1 | 1 | 1 | 1 |
| *VvGA20ox2* | 2 | 1 | 2 | | — | 2 | 2 | — | — |
| *VvGA20ox3* | — | 2 | 2 | | — | — | 3 | — | — |
| *VvGA20ox4* | 4 | 1 | 1 | | — | 1 | 1 | 1 | — |
| *VvGA20ox5* | — | — | 1 | | — | 1 | — | 1 | — |
| *VvGA20ox6* | 2 | 1 | 2 | | 1 | 2 | 1 | — | — |
| *VvGA20ox7* | 4 | — | — | | 1 | — | — | — | — |
