# Supplementary figures and images for: Genome-Wide Identification and Expression Analysis of GA2ox, GA3ox, and GA20ox Are Related to Gibberellin Oxidase Genes in Grape (Vitis vinifera L.)
Source: Genes (Basel). 2019 Sep 5;10(9):680. doi: 10.3390/genes10090680 (PMC6771001; doi:10.3390/genes10090680)

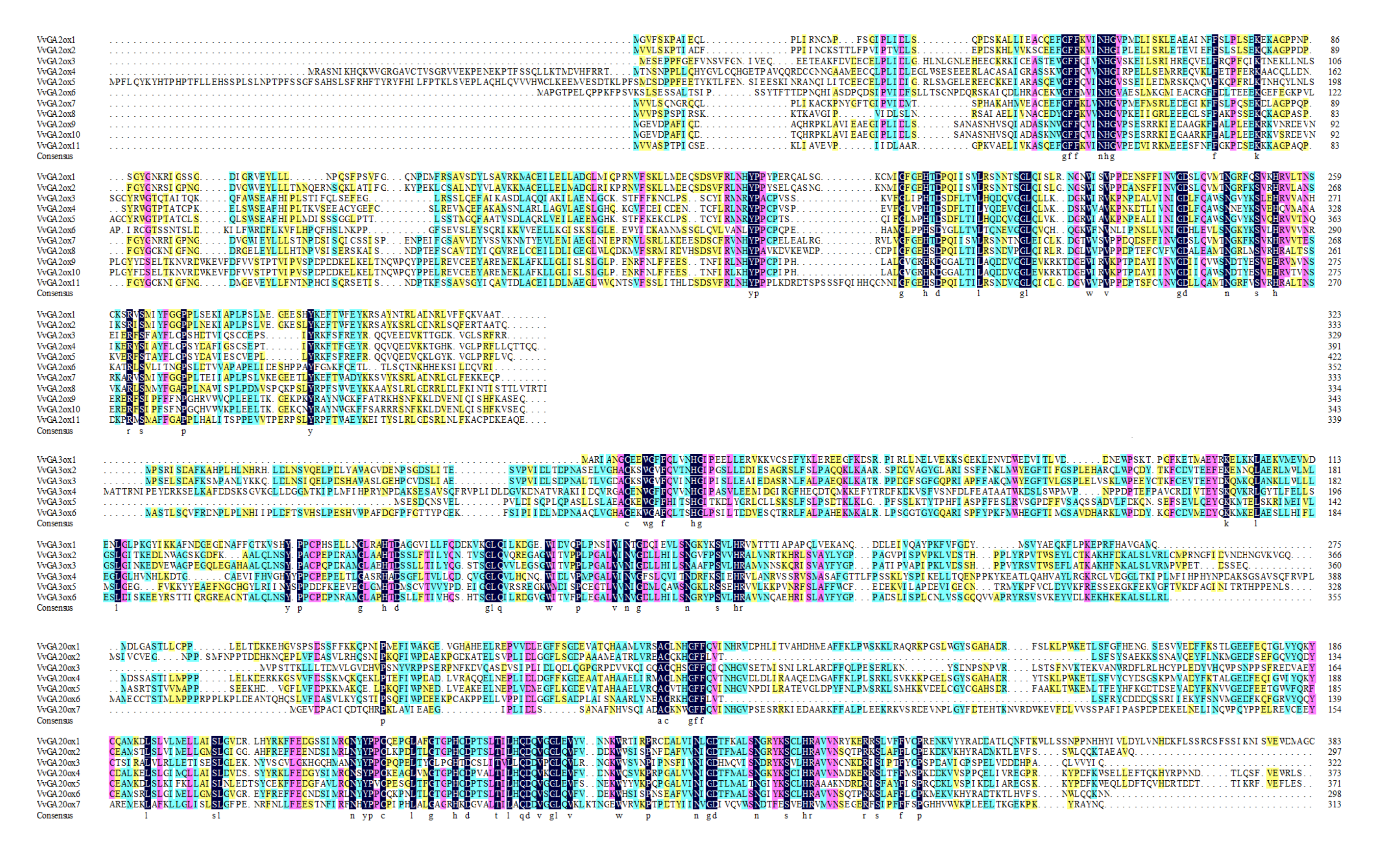

Supplement: Supplementary file 1 [file genes-10-00680-s001.zip › Fig S2.png]

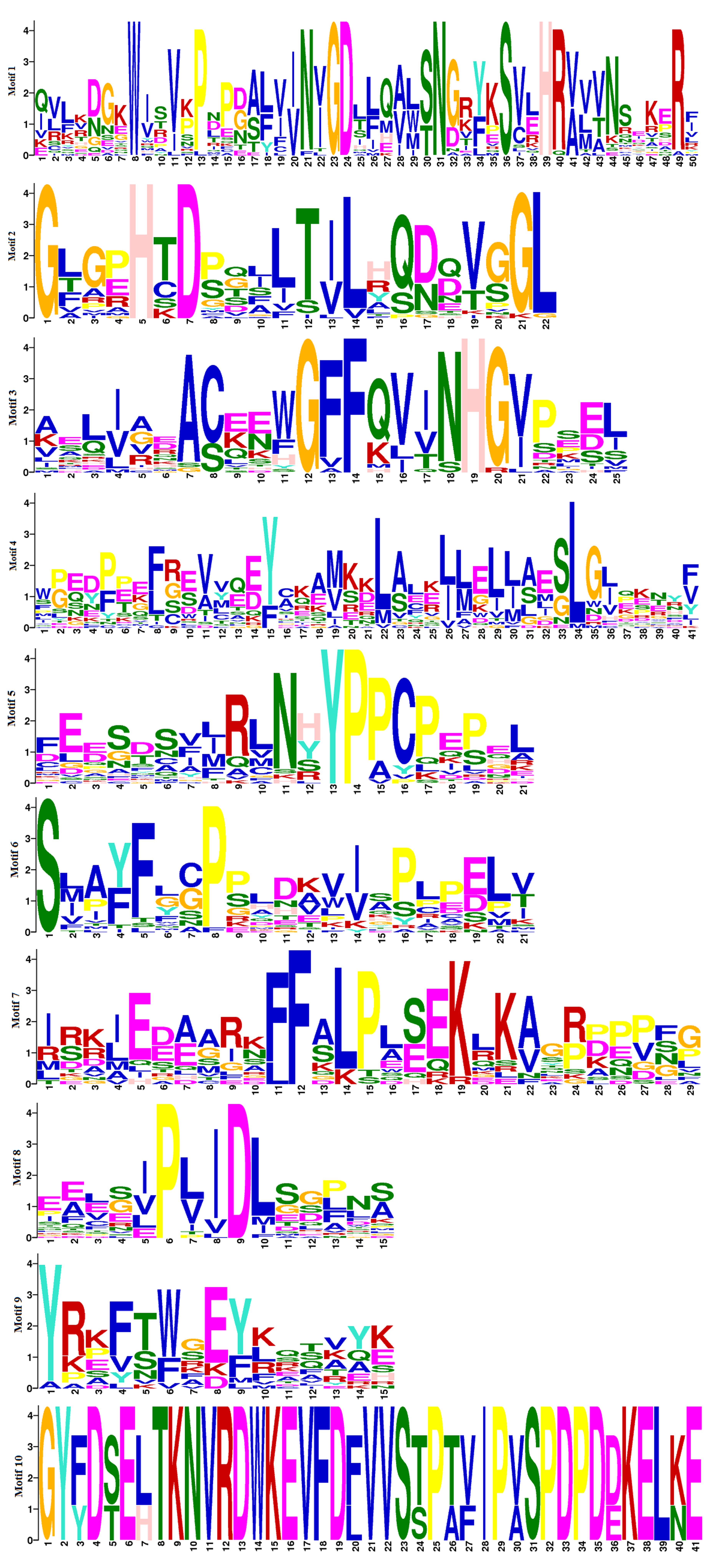

Supplement: Supplementary file 1 [file genes-10-00680-s001.zip › Fig S3.png]
